# Supplementary material for: Are carbon emissions trading and green financial instruments synergistic? -Comprehensive quantitative research based on content analysis
Source: PLoS One. 2024 Mar 7;19(3):e0298601. doi: 10.1371/journal.pone.0298601 (PMC10919601; doi:10.1371/journal.pone.0298601)
Supplement: S1 Data — (ZIP) [file pone.0298601.s001.zip › Data/地级市Z_F工作报告.zip!/║╙─╧/╚2├┼╧┐╩╨/└┤╫╘╣1⁄2╓┌║┼í╛┬φ┐╦╩2╛▌═°í┐_2006.docx]

各位代表：

　　现在，我代表市人民政府向大会作政府工作报告，请予审议，并请各位政协委员和其他列席人士提出意见。

　　一、“十五”及2005年工作回顾

　　“十五”以来，在省委、省政府和市委的正确领导下，在市人大、市政协的监督支持下，市政府团结带领全市人民，紧紧围绕全面建设小康社会的宏伟目标，坚持抓好发展第一要务，强力推进工业化、城镇化和农业现代化，综合经济实力明显增强，各项社会事业全面发展，正在稳步跨入中原崛起第一梯队。据初步测算，“十五”期间，全市生产总值年均增长11%，“十五”末人均突破15000元；地方财政一般预算收入年均增长15.7%。城镇居民人均可支配收入与农民人均纯收入比“九五”末分别增加2855元和789元。五年全社会固定资产投资累计完成475亿元，比“九五”时期增长1倍。利用外商直接投资2.2亿美元，增长1.27倍；出口创汇4.6亿美元，增长6.7倍。经济结构更趋合理，三次产业比例由“九五”末的13.4:52.4:34.2调整到9.1:61.5:29.4；高新技术产业增加值占工业增加值的比重达到9.3%，提高5.8个百分点。城镇化率38.8%，提高5.9个百分点。总体来看，“十五”计划目标全面或超额完成，为“十一五”发展奠定了坚实的基础。

　　刚刚过去的2005年，是我市“十五”期间经济社会发展速度最快、质量最好的一年，是改革开放和现代化建设取得显著成就的一年。全市上下认真落实科学发展观，牢牢把握“加快发展，维护稳定，狠抓落实”的工作大局，团结一致，开拓创新，圆满完成了市四届人大六次会议提出的工作任务，全面实现了会议确定的主要预期目标。预计全市生产总值达到340亿元，比上年增长14.5%。

　　项目建设突飞猛进，固定资产投资增势强劲。全社会固定资产投资预计完成170亿元，项目建设呈现出你追我赶、竞相发展的喜人局面。年初确定的“421”工程超额完成：投资在3000万元以上的工业项目达到93个，建成投产30个，新开工45个。“三大项目”建设进展顺利，大唐三门峡发电公司一号机组、东方希望氧化铝一期工程即将投入运行，英国开曼氧化铝一期工程建成投产。

　　支柱产业不断壮大，五大基地初具规模。全面加快能源、煤化工、铝工业、黄金、果品生产及加工基地建设步伐，产能迅速增加，五大支柱产业增加值占全市工业增加值的比重达到86%。预计全市规模以上工业完成增加值140亿元，比上年增长20%；经济效益综合指数180%，提高27.6个百分点；实现利税47.8亿元，增长35%。

　　城市建设力度加大，城镇化水平显著提升。积极实施中心城市带动战略，《三门峡市城市总体规划》批准实施。加快城市基础设施建设步伐，新修五原东路、文化南路、大岭南路，涧河四期治理初显形象，陕州大道两侧和涧河公园基础绿化基本完工，涧南城区改造取得较大进展，台下区如期实现集中供热，集中供气管网覆盖率达到60%。加快推进城镇化，310国道沿线城镇经济隆起带得到较快发展，经济总量达到全市

　　的75%。大力进行城市环境综合整治，市区空气质量明显改善。深入开展“四城联创”活动，顺利通过省级卫生城市复检和园林城市验收，入选“2005年度中国特色魅力城市200强”。

　　农业“三化”步伐加快，农村经济取得新进展。抓好农产品基地建设，建成36万亩无公害苹果、100万头牲畜、25万亩烟叶和68万亩速生丰产林基地。果、牧、林三大支柱产业发展壮大，预计全市果品产量110万吨，肉类产量7万吨，奶类产量1.4万吨。农业产业化快速发展，140余个龙头企业辐射带动农户26.8万户，实现销售收入12.6亿元。农业标准化和信息化建设日益完善，农产品检测网络覆盖城乡，“三电合一”信息化经验受到农业部表彰并在全国推广，烟叶生产全国领先。大力发展劳务经济，转移农村劳动力18万人，实现收入11亿元。

　　各项改革稳步推进，对外开放不断扩大。52家国有工业企业完成改制48家，灵宝黄金股份公司即将在香港上市。乡镇机构改革全面完成，粮食、流通、农信、文化、交通等改革稳步实施。开放型经济迅速发展，预计全年实际利用外资7208万美元，比上年增长25.2%；出口创汇1.36亿美元，增长12.4%。特色旅游日趋活跃，预计接待国内外游客318.7万人次，增长19%；实现旅游收入21.6亿元，增长11%。非公有制经济增势迅猛，全年新上项目235个，预计完成增加值113亿元，增长23%；入库税金9.7亿元，增长35%。

　　财政金融平稳运行，县域经济实力增强。财政收支取得突破性进展，地方财政一般预算收入完成16.6亿元，比上年增长39.5%，总量和增幅分别位居全省第9位和第6位；支出完成31.6亿元，增长35.5%。金融机构各项存款余额274亿元，增长21.5%；各项贷款余额199.2亿元，增长9.7%。县域经济迅速发展壮大，预计县域生产总值完成285亿元，增长17%，占全市总量的84%。渑池、灵宝财政收入超3亿元，义马、陕县超2亿元，卢氏超1亿元。

　　社会事业全面进步，人民生活水平明显改善。大力推进自主创新，预计全市专利申请量完成205件，比上年增长64%。农村教育稳步发展，提前一年完成D级危房改造任务，“两免一补”工作在全省名列前茅。成功举办了市五运会，群众性体育活动蓬勃开展。公共卫生体系建设力度加大，县级疾控项目和34所乡（镇）卫生院改造全部完成。计生工作不断加强，人口自然增长率4.03‰。“村村通”工程完成710公里，新增通油路行政村262个。实施扶贫项目148个，解决了1.6万人的温饱问题和52个行政村的人畜饮水困难。1.8万名下岗失业人员实现再就业，“两个确保”达到100%，城市低保对象实现应保尽保。“清欠”工作走在全省前列。预计全市城镇居民人均可支配收入完成8100元，增长12%；农民人均现金收入完成3220元，增长18.9%。民族宗教、妇女儿童工作被评为全国先进，实现省级“双拥模范城”五连冠。审计、物价、外侨、对台、农机、气象、人防、档案、史志、无线电、广播电视、新闻出版、河务移民、防震减灾和残疾人事业等取得新进展。

　　精神文明和民主法制建设得到加强，构建和谐社会有序推进。深入开展“三好一强”和保持共产党员先进性教育活动，“双创”和“两抓一促”效果明显。严格执行市人大及其常委会通过的决定、决议，办理人大代表建议50件、政协委员提案280件。坚持依法治市，基层执法队伍得到加强，“三严一创”活动有序推进，治安防控体系不断完善，荣获全省、全国社会治安综合治理优秀城市。认真落实党风廉政建设责任制，反腐纠风取得实效。开展以小煤矿为重点的专项整治，安全形势趋于平稳。深入贯彻实施《信访条例》，集中处理信访案件，保持了社会大局稳定。

　　这些成绩的取得，得益于积极落实中央、省宏观调控政策和市委确定的在调控中加快发展、在发展中顺应调控的工作方针；得益于突出发展第一要务，坚持工业的核心主导地位不动摇，大力实施“扩大开放，依托提升，优化结构，项目带动”的发展战略；得益于市人大、市政协的大力支持，全市上下同心协力，社会各界团结一致，形成思发展、谋发展、促发展的浓厚氛围。值此，我代表市人民政府，向工作在各条战线上的广大工人、农民、知识分子、干部、人民解放军指战员、武警官兵以及社会各界人士致以崇高的敬意和衷心的感谢！

　　在总结成绩的同时，我们也清醒地看到，经济社会发展进程中还存在不少困难和问题：一是产业结构不尽合理，资源开发和初加工比例偏高，高新技术产业比重较低，服务业发展比较滞后。二是农业综合生产能力提高不快，农民持续增收的难度依然较大，建设社会主义新农村的任务还很艰巨。三是中心城市辐射带动能力较弱，综合竞争力有待于逐步提高。四是政府职能转变还不能适应新形势的要求，依法行政和作风建设需要进一步加强。我们一定高度重视这些问题，采取有力措施，切实加以解决。

　　二、2006年及“十一五”时期政府工作的

　　总体要求、基本原则和预期目标

　　根据中央和省委经济工作会议以及市委四届六次全会精神，确定今年乃至“十一五”时期经济社会发展的总体要求是：以邓小平理论和“三个代表”重要思想为指导，以科学发展观统领经济社会发展全局，紧紧围绕跻身中原崛起第一梯队和提前实现全面建设小康社会的奋斗目标，突出加快发展与构建和谐社会两大任务，牢牢把握“加快发展，维护稳定，狠抓落实”的工作大局，大力实施“扩大开放，依托提升，优化结构，项目带动”发展战略，优化经济结构，转变增长方式，增强自主创新能力，奋力推进工业化、城镇化和农业现代化，努力实现又快又好的发展，切实推动经济社会转入科学发展的轨道。

　　按照上述总体要求，在工作中要始终坚持六个基本原则：

　　坚持突出第一要务。聚精会神搞建设，一心一意谋发展，竭尽全力抓落实，加压奋进，创优争先。

　　坚持转变增长方式。大力调整产业结构，强力推进自主创新，加快速度，改善质量，提高效益，实现全面协调可持续发展。

　　坚持推进城镇化。统筹规划，科学实施，突出产业支撑，强化辐射带动，以工促农，以城带乡，互动发展。

　　坚持抓好“三农”工作。全面落实政策，统筹城乡发展，加快推进“三化”，千方百计增加农民收入，建设社会主义新农村。

　　坚持加快改革开放。大力深化各项改革，努力扩大对外开放，创新机制，激发活力，以改革促开放，以开放促发展。

　　坚持构建和谐社会。牢记以人为本，更加关注民生，营造民主法治、公平正义、诚信友爱、充满活力、安定有序、人与自然和谐相处的社会环境。

　　《三门峡市国民经济和社会发展第十一个五年规划纲要（草案）》及其说明已印发各位代表，请予审议。

　　根据“十一五”规划确定的战略任务，今年国民经济和社会发展的主要预期目标是：生产总值增长13%；全社会固定资产投资增长20%；社会消费品零售总额增长14%；吸收外商直接投资增长20%，出口创汇增长16%；地方财政一般预算收入增长15%；城镇居民人均可支配收入增长9%，农民人均纯收入增长6%；万元生产总值能耗降低4.4%；人口自然增长率控制在6‰以内。

　　三、2006年的主要任务

　　为确保今年主要预期目标的实现，重点抓好以下七个方面的工作：

　　（一）坚定不移地推进重点项目建设，备足经济发展后劲

　　集中精力抓好在建项目，确保尽快投产一批。突出抓好“四大项目”，大唐三门峡发电公司二期两台机组上半年全部投产，同步建成50万伏输电线路；英国开曼、东方希望两大氧化铝二期工程按期完成投资计划；开祥化工100万吨甲醇一期25万吨项目年底前建成。千方百计加快进度，争取义马气源厂二期扩建、渑池天基5万吨铝型材、天鹏铝业高精度铝箔、陕县专用汽车装配生产线、灵宝新凌铅业10万吨电解铅、卢氏夜长坪钼矿二期改造等12个项目年内建成，形成新的经济增长点。

　　扎实推进前期工作，确保开工一批。抢抓机遇，创造条件，确保开祥化工5万吨1,4—丁二醇、义马10万吨二甲醚、义煤集团二期180万吨干法水泥、韶星氟化盐扩建、陕县捷马公司聚氯乙烯、灵宝5000吨高档铜箔、卢氏鸡湾电站、湖滨风力发电、开发区润德汽车座垫等30个项目年内相继开工，掀起新的投资高潮。

　　精心搞好项目库建设，确保储备一批。围绕“十一五”规划和“五大基地”建设的要求，筛选出一批事关我市经济长远发展的大项目和高新技术项目，补充完善各级项目库。重点抓好义马4×60万千瓦坑口电厂一期、陕县惠鑫铝业200万套车轮、湖滨20万吨离子膜烧碱、灵宝林纸一体化等一批重大项目的前期工作，争取实现大的突破，为“十一五”发展备足后劲。

　　（二）坚定不移地抓好产业结构的战略性调整，进一步提升产业整体竞争力

　　扶大做强支柱产业。围绕五大支柱产业，依托现有优势，大力实施名牌战略，努力打造一批在全省、全国有一定影响力的知名品牌，提升支柱产业的整体实力。能源工业，加快12个在建电力项目建设，年底电力装机达到450万千瓦。积极发展风电、水电等再生能源，壮大能源基地规模。铝工业，抓好铝土资源的利用、氧化铝的就地转化和电解铝的深加工，争取形成氧化铝200万吨、氢氧化铝10万吨、汽车轮毂300万只、铝型材7万吨的产能，建设全省最大的铝工业基地。煤化工工业，加快推进煤化工系列开发项目建设，加快形成45万吨甲醇、5万吨清洁油、5万吨1,4—丁二醇、6万吨苯酚等化工产品的产能。有色金属工业，立足当地，向外扩张，狠抓黄金、银、铜、铅等系列开发和精深加工以及伴生资源的综合利用，切实提高附加值，建设全国重要的黄金生产加工基地。林果业，巩固浓缩果汁产能，积极发展果胶、果糖、果酱、果渣饲料及其他果制品加工，新增果品精加工能力25万吨。壮大林板一体化规模，中高档密度板达到40万立方米。同时，依托医药产业基础，通过基地带动产业，重点开发渑池6万吨中药饮片和玫瑰油制品、豫西药业连蒲双清胶囊和盐酸纳美芬等系列产品，促进医药产业发展。

　　加快调整工业结构。围绕经济转型，抓好主导产业延伸，拉长产业链条，扩大终端产品生产，反复加工增值，实现产品结构由初级粗加工型向高级多元化转变，工业结构由资源开发向加工制造业转变。大力发展循环经济，抓好义马市循环经济试点，推动全市资源开发向综合利用、循环利用转变。积极发展生物工程、医药等高新技术产业，新建3—5家规模大、附加值高、市场前景好的高新技术企业，形成多点支撑的产业格局。

　　搞好工业园区建设。要认真搞好调研论证，与编制城市详细规划相结合，全面完善基础设施，适时启动三门峡西工业园建设。把工业园区作为产业基地和招商引资的重要平台，引导产业聚集。各县（市、区）要以现有布局为基础，以310国道沿线为重点，加快产业聚集，形成以产业基地为主导的“带状经济”。

　　大力发展服务业。围绕区域物流中心建设和强化现代城市服务功能的目标，规划物流网络布局。完善市场体系，抓好灵宝、渑池全国农村市场建设“万村千乡”试点工程，开拓农村市场。全面实施豫西文化体育长廊工程，推动广电数字进程，促进文化产业发展。积极发展金融、信息、会计、律师、仲裁、咨询等服务业，提升社区服务水平，加快形成辐射黄河金三角地区的流通、信息和现代服务业中心。

　　（三）坚定不移地抓好“三农”工作，全面建设社会主义新农村

　　加快建设社会主义新农村。按照“生产发展、生活宽裕、乡风文明、村容整洁、管理民主”的要求，科学规划，合理布局，稳步实施，确保“一年有突破，三年见成效，五年大变样”。认真贯彻“只予、不取、放活”的方针和一系列惠农政策，落实对种粮农民直接补贴、良种补贴和农业机械购置补贴，扩大公共财政在农村的覆盖面。强化农村信用社、供销社支农力度，进一步加大对农业、农村的投入，建立健全支农资金稳定增长机制。全面推进农村综合配套改革，进一步减轻农民负担，充分调动农民发展生产的积极性，努力实现农村经济有新发展，农民生活有新改善，农村容貌有新变化，管理水平有新提高。

　　强力发展现代农业。优化农业结构，做强优势产业，新建无公害优质苹果基地10万亩，新发展以高酸苹果为主的加工专用果5000亩。新建高标准养殖小区10个，新发展规模场20个、专业村15个，畜产品加工能力达到2万吨。深化林业产权制度改革，完成人工造林24万亩，飞播造林4万亩，通道绿化180公里。落实烟水配套政策，提高烟叶生产质量。果牧林烟等支柱产业当年产值要达到农业总产值的80%以上。大力发展农民专业合作组织，继续开展农业龙头企业“20强”竞赛活动，不断提高农业组织化、产业化水平。加强农业标准化、信息化建设，巩固完善“两级三层”农产品安全体系，提升信息服务质量。大力发展劳务经济，完成农村劳动力转移培训10万人次，有序转移20万人次，实现劳务收入12亿元。实施“科技入户”工程，完成农民科技培训15万人，先进实用技术入户率达到90%以上。

　　加强农村基础设施建设。以落实省委、省政府“十件实事”为着力点，全面改善农村生产生活条件。加快省道和“村村通”建设步伐，省道318、314、322市域段全部建成通车，“村村通”工程大头落地。加快卫家磨水库建设步伐，确保年底主体完工。完成水土流失治理面积80平方公里，新发展节水灌溉面积3万亩。积极实施改路、改水、改厕等生态家园建设，下大力气抓好农村沼气示范推广，控制农村面源污染，建立生态农业模式。切实搞好卢氏、灵宝、陕县农村新型合作医疗试点，加强乡镇卫生院建设，着力解决农民“看病难”问题。安排专项资金，完成610户、2362人的扶贫搬迁，解决1.6万贫困人口温饱和9.7万农村人口的饮水安全问题。建立健全农村教育、卫生、就业、社会保障等公共服务体系，完善动物疫病防控机制，加强自然灾害监测预报，提高民居防震抗灾能力，建立完善社会扶贫救助体系，努力实现“四有一不”救灾目标。

　　（四）坚定不移地实施中心城市带动战略，加快城镇化建设步伐

　　全面实施《三门峡市城市总体规划》。抓紧编制城市重点地区控制规划、义马渑池对接规划和城镇带建设等专项规划，切实提高城市规划管理水平。以调整区域中心城市行政区划、理顺城市规划管理体制为重点，严格三门峡市东西两区和开发区规划控制，自觉维护规划的严肃性，确保城市建设有序推进。

　　全力抓好城市基础设施重点工程。以创建国家园林城市为目标，加快区域中心城市建设，着重抓好涧河四期治理、涧南城区和“城中村”改造、陕州大道建设、天鹅湖景区完善提高、上阳苑山顶公园、小区街心绿地建设、甘棠北路和虢国西路新建、城市污水管网改造等基础设施工程。加快城市引水工程建设，改造、扩建市区供水管网，继续实施南山北岭大环境和城市道路、景区绿化亮化工程，提高城市绿化覆盖率，提升城市品位，打造“生态城市”。扎实抓好交通重大基础设施项目建设，争取临（汾）三（亚）高速公路三门峡段早日动工，全力配合搞好郑（州）西（安）快速客运专线工程建设。

　　积极推进310国道沿线城镇带建设。强化区域中心城市、副中心城市和重点镇的辐射带动作用，加快沿线乡镇建设步伐，逐步形成布局合理、功能健全、特色鲜明、组合有序的城镇体系。搞好义马城乡一体化试点。进一步强化乡镇产业支撑，鼓励因地制宜发展特色经济，培育新的增长点，把城镇带建成“工业走廊”，形成经济隆起带。

　　快速发展县域经济和非公有制经济。进一步落实和完善支持县域经济发展的政策措施，突出抓好义马的能源、煤化工，渑池的煤电铝一体化、建材、食品，陕县的热电、化工，灵宝的黄金、化工、林果生产加工，卢氏的绿色食品、矿业、旅游等，做大主导产业，增强县域经济的竞争力和综合实力。坚持把促进非公有制经济发展作为经济工作的重点，放宽市场准入，落实政策措施，建立激励机制，完善融资担保、创业辅导等服务体系，鼓励私营业主扩大再生产，力争非公有制经济增加值增长15.5%，入库税金增长13%。

　　下大力气改善环境质量。按照国家环保模范城市的要求，以市区大气污染、市区至大坝公路两侧、高速公路沿线及旅游景区环境污染综合整治为重点，深入开展电厂脱硫、机动车尾气治理、工业企业废水达标排放等专项行动，继续推进城市集中供热、集中供气工程，促进市区环境状况明显改善，全年大气质量二级以上天数达到80%以上。认真抓好市县两级污水处理厂、垃圾处理场建设，争取年底全部建成投入使用。逐步清理整顿砖瓦窑场，积极推广新技术、新工艺、新材料，实现城乡建筑节能降耗。

　　（五）坚定不移地推进各项改革，强力发展开放型经济

　　继续深化各项改革。继续加大国企改革力度，未改制企业要抓紧实施；帮助转制企业按新机制运行，焕发生机和活力。全力抓好流通企业改革，造就一批具有较强竞争能力的商贸和物流集团。加快经营性文化事业单位转企改制步伐，鼓励参与市场竞争。改革投融资体制，加快组建三门峡市商业银行。稳步推进城市公用行业改革，拓宽城市建设融资渠道。深化交通体制配套改革，加快公路事业发展。理顺县乡关系，促进乡镇政府转变职能，巩固乡镇机构改革成果。

　　全面扩大对外开放。大力实施开放带动战略，坚持对内对外开放相结合，充分利用两个市场、两种资源，发挥比较优势，全面发展开放型经济。进一步拓宽引资领域和规模，改进招商方式，明确招商主体，以铝产品深加工、煤化工、改造传统制造业等为重点筛选发布招商项目，引导境外资金投向重点区域、重点产业和重点企业，提高招商实效。优化出口商品结构，着力培育新的出口产品，扩大轮毂、精密量仪、纺织器材等机电产品和果汁等特色农产品出口，形成多元化的出口格局。继续实施东引西进，广泛开展战略合作，鼓励优势企业到市外建立产业基地，借助外部资源，拓宽发展空间。

　　培育提升特色旅游业。坚持把旅游业作为国民经济的支柱产业来培育，修编完善旅游业发展总体规划，认真落实发展旅游业的政策措施，营造支持旅游、发展旅游、服务旅游的良好氛围。围绕打造三门峡“文化山水”品牌，加快文化产业和生态旅游的有机结合，加强函谷关古文化旅游区、天鹅湖景区、豫西和仰韶两大峡谷、玉皇山国家森林公园等精品线路和景区建设，借势搞好旅游产品包装和品牌培育，精心策划在中央媒体的宣传促销，全力办好第十二届三门峡国际黄河旅游节暨投资贸易洽谈会。

　　扎实做好财政金融工作。深化财政体制改革，建立完善规范、科学的财政体制。依法加强税收征管，积极组织收入，确保应收尽收。切实加强预算管理，调整优化支出结构，保工资、保稳定、促发展，保障“三农”、教育、科技等重点支出需要。加快建立全市统一的信用信息基础数据库，继续做好中小企业信用评审工作，严厉打击逃废银行债务行为，改善金融生态环境。积极搭建银企合作平台，建立银企互动共赢的长效机制。依法加强金融监管，规范市场秩序，维护金融稳定。

　　（六）坚定不移地发展各项社会事业，不断提高人民生活水平

　　坚持实施可持续发展、科教兴市和人才强市“三大战略”。正确处理经济社会发展与人口、资源、环境的关系。理顺土地管理体制，实现耕地占补平衡。落实“责任、措施、投入”三到位，稳定低生育水平，提高出生人口素质。加强环境执法，强化资源节约和生态保护，建设环境友好型和资源节约型社会。充分发挥科技支撑作用，大力推进科技进步与自主创新。进一步整合优化教育资源，加大教育投入，改善办学条件，提高教师待遇，稳定教师队伍，创建1—2所省级示范性高中、3—5所市级示范性初中、10所以上市级示范性小学和幼儿园。继续落实好“两免一补”政策，解决贫困学生入学问题。强化素质教育，大力发展职业教育和成人教育。创新人才工作机制，加大人才引进力度和人事管理信息化建设，促进人才合理流动。

　　扎实做好就业和社会保障工作。继续完善鼓励就业和再就业的各项政策措施，建立扩大就业的有效机制，努力提高失业人员的再就业率。积极开发公益性岗位，安排“4050”人员就业，认真做好大中专毕业生就业、复员转业军人安置工作。进一步完善社会保障和城镇困难群体救助体系，提高城市低保标准；扩大社会保险覆盖面，确保企业离退休人员养老金按时足额发放；拓展农村低保试点范围，建立健全与经济发展水平相适应的社会保障制度。

　　切实加快社会事业发展。抓好疾病预防控制体系和医疗救治体系建设，市疾病预防控制中心、市卫生监督中心和市第二人民医院投入运行，提高应对突发公共卫生事件的能力。改善医疗服务质量，认真解决“看病贵”问题。积极发展优势竞技体育项目，广泛开展群众性体育活动，不断增强人民体质。加强国防教育、人防工作和民兵预备役建设，推进双拥和军民共建活动，争取全国“双拥模范城”四连冠。进一步做好外侨、民族、宗教、残疾人、老龄、档案、史志、气象、无线电等工作。

　　（七）坚定不移地抓好“三大文明”建设，努力构建和谐社会

　　加强社会主义精神文明建设。深化“两抓一促”工程，继续开展“道德规范进万家，诚实守信万人行”活动，强力推进“双创”和文明单位、文明村镇、文明社区创建活动，搞好精神文明基础设施建设。大力弘扬民族精神、时代精神和科学精神，倡导文明健康的生活方式，净化未成年人成长环境。把握正确的舆论导向，繁荣和发展文学艺术及新闻出版事业。加强文化市场管理，深入开展“三下乡”活动，活跃农村、社区群众文化生活，满足人民群众日益增长的精神文化需求。

　　加强社会主义民主政治建设。自觉接受人大及其常委会的监督，认真落实决议、决定，及时报告工作。积极支持人民政协履行政治协商、民主监督、参政议政职能，主动听取社会各界的意见和建议。认真办理好人大代表议案、建议和政协委员提案，完善科学民主决策机制，提高决策透明度和公众参与度。切实加强与各民主党派、工商联和无党派人士的联系，充分发挥工会、共青团、妇联等人民团体的桥梁纽带作用，扩大基层民主，保障人民群众的民主政治权利。

　　加强社会主义法制建设。坚持依法治市，加大“五五”普法力度，增强全民法制意识。围绕建设“平安三门峡”，进一步加强社会治安综合治理，深入开展“三严一创”活动，依法严厉打击各种刑事犯罪和邪教组织的破坏活动，维护社会稳定。认真落实安全生产责任制，健全安全生产监管体制，突出抓好矿山、交通运输、危险化学品、公众聚集场所等重点行业、重点领域的安全生产专项整治，坚决遏制重特大事故发生。继续推进“下访包案”，排查矛盾，化解纠纷，认真解决群众反映的问题，为经济发展营造稳定和谐的社会环境。

　　四、努力建设服务型政府

　　牢记全心全意为人民服务的根本宗旨，按照“为民、务实、清廉”的要求，切实加强政府自身建设，提高执政能力，努力做到为人民服务，对人民负责，受人民监督，让人民满意。

　　（一）切实转变政府职能。正确把握政府职能定位，完善和强化政府的经济调节、市场监管、社会管理和公共服务职能，以人为本，激发活力，以政府职能转变促和谐社会建设。加强煤炭、电力、天然气、运力等生产要素的供求衔接，强化经济运行调节，提高保障能力。进一步深化行政审批制度改革，优化经济发展环境，拓展阳光服务平台。发展电子政务，加强政务公开，提高服务质量和效率。加快建立健全各种突发事件应急机制，全面提高保障公共安全和处置突发事件的能力。广大公务员要注重学习和把握国家的经济方针、产业政策，结合自身工作，超前思考，准确理解，提高驾驭市场经济和解决复杂问题的能力。

　　（二）切实提高决策执行能力。进一步健全制度，规范程序，明确责任，

　　推进决策科学化、民主化。对关系经济社会发展全局和涉及公共利益的

　　重大问题，要认真进行专家论证和评估，履行公示、听证等民主程序，

　　积极征询各方面意见，形成有利于广泛集中民智、反映民意的决策机制。简化工作流程，强化绩效管理，狠抓工作落实，严格责任追究，坚决杜

　　绝推诿扯皮、中梗阻塞的现象，确保政令畅通。要分清轻重缓急，明确目标责任，集中精力抓大事，竭尽全力办好事。

　　（三）切实推进依法行政。深入贯彻实施《行政许可法》和《国务院全面推进依法行政实施纲要》，把政府管理工作纳入规范化、法制化轨道。严格实行行政执法责任制和过错追究制。加强行政监察和审计工作，深入开展廉政建设和反腐败斗争，建立健

　　全教育、制度、监督并重的预防和惩治腐败体系。政府全体工作人员要常修为政之德，常思贪欲之害，常怀律己之心，奉公守法，勤政廉政，当好公仆。

　　（四）切实改进工作作风。牢固树立科学发展观和正确政绩观，大兴求真务实之风，坚持一切从实际出发

　　，力戒急功近利，力戒形式主义，力戒官僚作风，多做打基础、备后劲、谋长远的工作，多办察民情、得民

　　心、顺民意的好事，经得起历史、经得起群众、经得起实践的检验。要始终保持奋发有为的

　　精神状态，进一步增强加快发展的紧迫感，激发干事创业的使命感，脚踏实地，不骄不躁，埋头苦干，以

　　加压奋进的勇气、创优争先的志气、开拓进取的锐气，确保政府各项工作事事落到实处，件件取得成效，不断创造新业绩，努力开创新局面，为实现“十一五”良好开局奠定坚实的基础。

　　各位代表，2006年是我们站在新的历史起点上、向更高目标迈进的一年。切实做好今年的工作，意义重大而深远，任务光荣而艰巨。让我们在以胡锦涛同志为总书记的党中央领导下，高举邓小平理论和“三个代表”重要思想伟大旗帜，认真贯彻党的十六届五中全会精神，全面落实科学发展观，解放思想，求真务实，扎实工作，开拓创新，为早日跻身中原崛起第一梯队和提前实现全面建设小康社会的宏伟目标而努力奋斗！
